# Supplementary material for: End Sequence Analysis Toolkit (ESAT) expands the extractable information from single-cell RNA-seq data
Source: Genome Res. 2016 Oct;26(10):1397–410. doi: 10.1101/gr.207902.116 (PMC5052061; doi:10.1101/gr.207902.116)
Supplement: Supplemental Material [file supp_gr.207902.116_Supp_Materials.tar.gz › ESAT-esat_v0.1/doc/html/classbroad_1_1pda_1_1feature_1_1genome_1_1_directory_installed_genome_assembly.html]

ESAT: broad.pda.feature.genome.DirectoryInstalledGenomeAssembly Class Reference


ESAT


- Main Page
- Related Pages
- Packages
- Classes
- Files

- Class List
- Class Index
- Class Hierarchy
- Class Members

All Classes Namespaces Files Functions Variables Enumerator Pages

- broad
- pda
- feature
- genome
- DirectoryInstalledGenomeAssembly

Public Member Functions |
Protected Member Functions |
Protected Attributes |
List of all members

broad.pda.feature.genome.DirectoryInstalledGenomeAssembly Class Reference

Collaboration diagram for broad.pda.feature.genome.DirectoryInstalledGenomeAssembly:

[legend]

|  |  |
| --- | --- |
| Public Member Functions | |
|  | DirectoryInstalledGenomeAssembly (File sequenceDirectory) throws Exception |
|  | |
| List< Chromosome > | getAllNonRandomChromosomes () |
|  | |
| List< Chromosome > | getAllChromosomes () |
|  | |
| Chromosome | getX () |
|  | |
| void | writeSizes (String outFile) throws IOException |
|  | |
| long | getNonRandomTotalSize () |
|  | |
| List< Chromosome > | getAutosomes () |
|  | |
| int | getAutosomeNumber () |
|  | |
| Chromosome | getChromosome (String chr) |
|  | |
| List< Chromosome > | getUn () |
|  | |
| long | getGenomeSize () |
|  | |
| long | getAutosomeGenomeSize () |
|  | |
| Chromosome | drawChromosome () |
|  | |
| SequenceRegion | drawRandomRegion (int size) |
|  | |
| Chromosome | drawAutosome () |
|  | |
| SequenceRegion | drawRandomAutosomeRegion (int size) |
|  | |
| File | getSequenceDir () |
|  | |
| File | getRepeatFile (String chrSymbol) |
|  | |
| File | getAnnotationDirectory () |
|  | |
| List< GenomicAnnotation > | getReducedRepresentation (int totalSize, List< GenomicAnnotation > toAvoid) |
|  | |
| void | delete (SequenceRegion region) |
|  | |
| void | invert (SequenceRegion region) |
|  | |
| void | insert (SequenceRegion region, GenomicAnnotation insertionPoint) |
|  | |
| void | translocate (SequenceRegion region1, SequenceRegion region2) |
|  | |

|  |  |
| --- | --- |
| Protected Member Functions | |
| boolean | areRepeatsInDeepDirHierarchy () |
|  | |
| boolean | useMergedUn () |
|  | |
| boolean | doRepeatListNeedsCleanup () |
|  | |

|  |  |
| --- | --- |
| Protected Attributes | |
| File | sequenceDirectory |
|  | |

## Detailed Description

Represents a Genome Assembly were each directory is in its own directory which includes the AGP file It is a convenient way to access genomic data when it is stored in this standard way.

Author
:   mgarber

## Constructor & Destructor Documentation

|  |  |  |  |  |  |
| --- | --- | --- | --- | --- | --- |
| broad.pda.feature.genome.DirectoryInstalledGenomeAssembly.DirectoryInstalledGenomeAssembly | ( | File | *sequenceDirectory* | ) | throws Exception |

Here is the call graph for this function:

## Member Function Documentation

|  |  |  |  |  |
| --- | --- | --- | --- | --- |
| boolean broad.pda.feature.genome.DirectoryInstalledGenomeAssembly.areRepeatsInDeepDirHierarchy | ( |  | ) |  |

protected

to be overrided if repeats are in directories

Returns

Here is the caller graph for this function:

|  |  |  |  |  |  |
| --- | --- | --- | --- | --- | --- |
| void broad.pda.feature.genome.DirectoryInstalledGenomeAssembly.delete | ( | SequenceRegion | *region* | ) |  |

Here is the call graph for this function:

|  |  |  |  |  |
| --- | --- | --- | --- | --- |
| boolean broad.pda.feature.genome.DirectoryInstalledGenomeAssembly.doRepeatListNeedsCleanup | ( |  | ) |  |

protected

subclasses should override if the repeats contain overlapping segments (i.e. are merged with trf)

Returns

|  |  |  |  |  |
| --- | --- | --- | --- | --- |
| Chromosome broad.pda.feature.genome.DirectoryInstalledGenomeAssembly.drawAutosome | ( |  | ) |  |

Uses pseudo random number generation to draw a (non random) autosome with chance proportional to the autosome size

Returns
:   Chromosome draw by random.

Here is the call graph for this function:

Here is the caller graph for this function:

|  |  |  |  |  |
| --- | --- | --- | --- | --- |
| Chromosome broad.pda.feature.genome.DirectoryInstalledGenomeAssembly.drawChromosome | ( |  | ) |  |

Uses pseudo random number generation to draw a (non random) chromosome with chance proportional to the chromosome size

Returns
:   Chromosome draw by random.

Here is the call graph for this function:

Here is the caller graph for this function:

|  |  |  |  |  |  |
| --- | --- | --- | --- | --- | --- |
| SequenceRegion broad.pda.feature.genome.DirectoryInstalledGenomeAssembly.drawRandomAutosomeRegion | ( | int | *size* | ) |  |

Here is the call graph for this function:

|  |  |  |  |  |  |
| --- | --- | --- | --- | --- | --- |
| SequenceRegion broad.pda.feature.genome.DirectoryInstalledGenomeAssembly.drawRandomRegion | ( | int | *size* | ) |  |

Here is the call graph for this function:

|  |  |  |  |  |
| --- | --- | --- | --- | --- |
| List<Chromosome> broad.pda.feature.genome.DirectoryInstalledGenomeAssembly.getAllChromosomes | ( |  | ) |  |

Here is the call graph for this function:

Here is the caller graph for this function:

|  |  |  |  |  |
| --- | --- | --- | --- | --- |
| List<Chromosome> broad.pda.feature.genome.DirectoryInstalledGenomeAssembly.getAllNonRandomChromosomes | ( |  | ) |  |

Here is the caller graph for this function:

|  |  |  |  |  |
| --- | --- | --- | --- | --- |
| File broad.pda.feature.genome.DirectoryInstalledGenomeAssembly.getAnnotationDirectory | ( |  | ) |  |

|  |  |  |  |  |
| --- | --- | --- | --- | --- |
| long broad.pda.feature.genome.DirectoryInstalledGenomeAssembly.getAutosomeGenomeSize | ( |  | ) |  |

Here is the caller graph for this function:

|  |  |  |  |  |
| --- | --- | --- | --- | --- |
| int broad.pda.feature.genome.DirectoryInstalledGenomeAssembly.getAutosomeNumber | ( |  | ) |  |

|  |  |  |  |  |
| --- | --- | --- | --- | --- |
| List<Chromosome> broad.pda.feature.genome.DirectoryInstalledGenomeAssembly.getAutosomes | ( |  | ) |  |

Here is the caller graph for this function:

|  |  |  |  |  |  |
| --- | --- | --- | --- | --- | --- |
| Chromosome broad.pda.feature.genome.DirectoryInstalledGenomeAssembly.getChromosome | ( | String | *chr* | ) |  |

Here is the call graph for this function:

Here is the caller graph for this function:

|  |  |  |  |  |
| --- | --- | --- | --- | --- |
| long broad.pda.feature.genome.DirectoryInstalledGenomeAssembly.getGenomeSize | ( |  | ) |  |

Here is the caller graph for this function:

|  |  |  |  |  |
| --- | --- | --- | --- | --- |
| long broad.pda.feature.genome.DirectoryInstalledGenomeAssembly.getNonRandomTotalSize | ( |  | ) |  |

Here is the call graph for this function:

|  |  |  |  |
| --- | --- | --- | --- |
| List<GenomicAnnotation> broad.pda.feature.genome.DirectoryInstalledGenomeAssembly.getReducedRepresentation | ( | int | *totalSize*, |
|  |  | List< GenomicAnnotation > | *toAvoid* |
|  | ) |  |  |

Here is the call graph for this function:

|  |  |  |  |  |  |
| --- | --- | --- | --- | --- | --- |
| File broad.pda.feature.genome.DirectoryInstalledGenomeAssembly.getRepeatFile | ( | String | *chrSymbol* | ) |  |

Here is the call graph for this function:

|  |  |  |  |  |
| --- | --- | --- | --- | --- |
| File broad.pda.feature.genome.DirectoryInstalledGenomeAssembly.getSequenceDir | ( |  | ) |  |

|  |  |  |  |  |
| --- | --- | --- | --- | --- |
| List<Chromosome> broad.pda.feature.genome.DirectoryInstalledGenomeAssembly.getUn | ( |  | ) |  |

|  |  |  |  |  |
| --- | --- | --- | --- | --- |
| Chromosome broad.pda.feature.genome.DirectoryInstalledGenomeAssembly.getX | ( |  | ) |  |

|  |  |  |  |
| --- | --- | --- | --- |
| void broad.pda.feature.genome.DirectoryInstalledGenomeAssembly.insert | ( | SequenceRegion | *region*, |
|  |  | GenomicAnnotation | *insertionPoint* |
|  | ) |  |  |

Here is the call graph for this function:

|  |  |  |  |  |  |
| --- | --- | --- | --- | --- | --- |
| void broad.pda.feature.genome.DirectoryInstalledGenomeAssembly.invert | ( | SequenceRegion | *region* | ) |  |

Here is the call graph for this function:

|  |  |  |  |
| --- | --- | --- | --- |
| void broad.pda.feature.genome.DirectoryInstalledGenomeAssembly.translocate | ( | SequenceRegion | *region1*, |
|  |  | SequenceRegion | *region2* |
|  | ) |  |  |

Here is the call graph for this function:

|  |  |  |  |  |
| --- | --- | --- | --- | --- |
| boolean broad.pda.feature.genome.DirectoryInstalledGenomeAssembly.useMergedUn | ( |  | ) |  |

protected

Here is the caller graph for this function:

|  |  |  |  |  |  |
| --- | --- | --- | --- | --- | --- |
| void broad.pda.feature.genome.DirectoryInstalledGenomeAssembly.writeSizes | ( | String | *outFile* | ) | throws IOException |

Here is the call graph for this function:

## Member Data Documentation

File broad.pda.feature.genome.DirectoryInstalledGenomeAssembly.sequenceDirectory

protected

---

The documentation for this class was generated from the following file:

- java/broad/pda/feature/genome/DirectoryInstalledGenomeAssembly.java


---

Generated on Fri Aug 15 2014 15:10:59 for ESAT by  

 1.8.7
